# Associations of subclinical autistic-like traits with brain structural variation using diffusion tensor imaging and voxel-based morphometry

**DOI:** 10.1192/j.eurpsy.2021.15

**Published:** 2021-03-03

**Authors:** Yvonne Schröder, Daniela Michelle Hohmann, Tina Meller, Ulrika Evermann, Julia-Katharina Pfarr, Andreas Jansen, Inge Kamp-Becker, Sarah Grezellschak, Igor Nenadić

**Affiliations:** 1 Cognitive Neuropsychiatry Lab, Department of Psychiatry and Psychotherapy, Philipps-University Marburg/Marburg University Hospital—UKGM, Marburg, Germany; 2 Center for Mind, Brain and Behavior (CMBB), Hans-Meerwein-Str. 6, 35032 Marburg, Germany; 3 Core-Facility BrainImaging, School of Medicine, Philipps University Marburg, Marburg, Germany; 4 Department of Child and Adolescent Psychiatry, Psychosomatics and Psychotherapy, Philipps-University Marburg/Marburg University Hospital—UKGM, Marburg, Germany

**Keywords:** Autism spectrum disorder, autism spectrum quotient, autistic traits, diffusion tensor imaging, voxel-based morphometry

## Abstract

**Background:**

Previous case–control studies of autistic spectrum disorder (ASD) have identified altered brain structure such as altered frontal and temporal cortex volumes, or decreased fractional anisotropy (FA) within the inferior fronto-occipital fasciculus in patients. It remains unclear whether subclinical autistic-like traits might also be related to variation in these brain structures.

**Methods:**

In this study, we analyzed magnetic resonance imaging (MRI) data of 250 psychiatrically healthy subjects phenotyped for subclinical autistic-like traits using the Autism Spectrum Quotient (AQ). For data analysis, we used voxel-based morphometry of T1-MRIs (Computational Anatomy Toolbox) and tract-based spatial statistics for diffusion tensor imaging data.

**Results:**

*AQ attention switching* subscale correlated negatively with FA values in the bilateral uncinate fasciculus as well as the bilateral inferior fronto-occipital fasciculus. Higher *AQ attention switching* subscale scores were associated with increased mean diffusivity and radial diffusivity values in the uncinate fasciculus, while axial diffusivity values within this tract show a negative correlation. *AQ attention to detail* subscale correlated positively with gray matter volume in the right pre- and postcentral gyrus.

**Conclusions:**

We demonstrate that individuals with higher levels of autism-spectrum-like features show decreased white matter integrity in tracts associated with higher-level visual processing and increased cortical volume in areas linked to movement sequencing and working memory. Our results resemble regional brain structure alterations found in individuals with ASD. This offers opportunities to further understand the etiology and pathogenesis of the disorder and shows a subclinical continuum perspective.

## Introduction

There is growing evidence that many of the traits and behaviors associated with autism spectrum disorder (ASD) are not restricted to those with a clinical diagnosis but also occur in other mental disorders [1,2] as well as in the general population [3]. There are different questionnaires to assess autistic-like traits in adults, most prominently the Autism Spectrum Quotient (AQ) [4]. The AQ is a self-report measure for autistic-like traits using a sum score as well as five different subscales, including *AQ social skills*, *AQ communication*, *AQ attention to detail*, *AQ attention switching,* and *AQ imagination* [4].

While patients with an ASD diagnosis show significantly higher scores than the general population, the AQ fails to differentiate ASD from other mental disorders [5,6]. Prior studies have found that autistic-like traits are continuously distributed across the population and the AQ is also widely used in nonclinical subjects [7]. Furthermore, studies were able to show that parents of children with ASD score higher on *AQ communication* and *AQ social skills* compared to parents of healthy children [8]. This indicates that autistic-like traits are also prevalent in nonclinical populations and that the AQ scores may show heritability in families just like autism itself, which is in line with the broader autism phenotype found in some parents of children with ASD [7].

Multiple studies have found that nonclinical individuals with higher AQ scores show impaired performance on different cognitive and behavioral tasks, with patterns resembling those of ASD patients. This includes global visual processing [9], reduced neural response to affective touch [10], or attention and emotion processing [11], as well as a preference for predictability [12]. With autistic-like traits being continuously distributed across the population, it is still unclear whether correlations with biological parameters such as in ASD patients are also present in nonclinical subjects to a lesser extent. Studying subclinical traits can enhance our understanding of the autism spectrum as it avoids limitations of disease course, chronicity, and medication, which commonly confound case–control studies.

Previous magnetic resonance imaging (MRI) studies also show that ASD patients exhibit brain abnormalities compared to healthy controls. Recent large-scale multicenter studies of the Enhancing Neuro Imaging Genetics through meta analysis (ENIGMA) consortium indicate smaller subcortical volumes in putamen, pallidum, and nucleus accumbens, as well as amygdala in ASD patients compared to healthy controls, with cortical thickness increases in frontal (esp. anterior lateral and medial) and decreases in temporal (esp. inferior and parahippocampal) cortical areas [13,14]. Using voxel-based morphometry (VBM), increased gray matter volume (GMV) in the left inferior temporal cortex was found in patients with autism compared to nonclinical, healthy subjects [15]. Other case–control studies have found that ASD patients had significantly increased GMV in the middle and superior temporal gyrus, as well as in the postcentral and parahippocampal gyrus while showing decreased GMV in the anterior cingulate cortex and cerebellum [16]. Yamasaki et al. (2017) [17] have suggested that the underlying pathophysiological mechanisms of ASD may constitute a “connectopathy” in parallel visual pathways and attention networks. Others criticize that while case–control studies very often show group differences in brain functional connectivity, patterns of findings vary considerably, which may be due to ASD and typically developing individuals differing systematically in response to resting state instructions and environments [18].

In contrast to ASD case–control studies, some initial studies suggest a correlation between nonclinical autistic-like traits and brain structural markers such as fractional anisotropy (FA) values in the left inferior longitudinal fasciculus [19] as well as in the inferior fronto-occipital fasciculus (IFOF) [20]. Another study regarding healthy adults showed a correlation between the volume of the white matter pathway between the superior temporal sulcus (STS) and amygdala (AMG) with the AQ total score [21]. A correlation for nonclinical autistic-like traits and GMVs was found in the left primary visual cortex [22] and the orbitofrontal cortex, cuneus, and (para)hippocampus in a high autistic trait sample [23]. A certain limitation of these previous samples is the analysis of only one MRI modality and in part also sample size (ranging from 24 to 91 individuals).

Understanding the etiology of neural correlations in autistic traits in the general population might aid our understanding of the causes of autism. However, to date, there is only a small number of studies to understand whether AQ scores of nonclinical subjects correlate with brain structural parameters, particularly in regions mirroring the results of clinical case–control studies of ASD. The present study tested the hypothesis that brain structural variation, particularly in regions affected by ASD, would also show a correlation with subclinical autistic-like traits (measured by AQ scores) in nonclinical individuals. We analyzed not only correlations of the AQ total score but also the subscales with biological parameters considering the heterogeneity of the phenotype to obtain facet-level associations. Similar to phenotype heterogeneity in autism spectrum disorders, these subscales represent manifestations of different autistic-like trait dimensions. Individuals may score high on one specific subscale but not the others. Therefore, correlations with biological parameters may be driven by a specific subscale, that is, autistic-like trait and thus represent specific biological mechanisms.

## Methods

### Study cohort

The overall sample of the study consisted of *N* = 250 nonclinical participants from the general population aged 18–40 years. Participants had a mean age of 23.9 years with a standard deviation (SD) of 3.9. The sample included 173 females (69.2%) and 77 males (30.8%). All participants were of European heritage and had a good understanding of the German language.

All participants had an intelligence quotient of 85 or higher estimated by a multiple-choice vocabulary test (Mehrfachwahl-Wortschatz-Intelligenztest) [24]. Handedness was assessed using the Edinburgh Handedness Inventory [25] resulting in a mean of 79.68 with an SD of 52.97. The distribution was as follows: 17 participants = left-handed (6.85%); 10 participants = ambidextrous (4.03%); 221 participants = right-handed (89.11%).

We screened all participants using the German version of the SKID-I, the structured clinical interview for DSM-IV Axis I Disorders, German version (Strukturiertes Klinisches Interview für DSM-IV Achse I Störungen) [26], to exclude a history of psychiatric disorders, substance abuse, and first-degree psychotic disorders in their families. Further exclusion criteria were major medical conditions, neurological conditions as well as medical contraindications for MRI such as pregnancy or nonremovable metal objects.

All subjects gave written consent before participating in the study. The study protocol was approved by the Ethics Committee of the Philipps University Medical School (protocol number 61/18) according to the latest version of the Declaration of Helsinki [27]. Financial compensation was given to the study participants.

### Phenotyping for autism-spectrum-like traits

We used the German version of the AQ in this study to measure the expression of autism-spectrum-like traits. The AQ is a widely established 50-item (long version) self-report measurement to evaluate the manifestation of autistic-like traits. It was first developed to measure the degree of autistic-like traits in clinical individuals with normal intelligence [4] but is also widely used in nonclinical population samples [7]. Previous studies show satisfying test–retest-stability for the AQ with *r* = 0.78 for the total AQ score and *r* = 0.60–0.81 for the subscales [28]. In our sample, internal consistency was assessed in the current study and was satisfactory for the *AQ total score* (Cronbach’s *α* = 0.717) as well as subscales *AQ social skills, AQ attention switching,* AQ *communication, and AQ imagination* and lower for the *AQ attention to detail* subscale: *AQ attention to detail* (Cronbach’s *α* = 0.373), *AQ social skills* (Cronbach’s *α* = 0.501), *AQ attention switching* (Cronbach’s *α* = 0.449), AQ *communication* (Cronbach’s *α* = 0.414), and *AQ imagination* (Cronbach’s *α* = 0.594). Since Cronbach’s *α* might be a suboptimal parameter for scales with less than 10 items, we additionally computed inter-item correlations [29], which are given in Supplementary Figures S1–S5.

### Data acquisition

We acquired MRI data using a Siemens Tim Trio 3 Tesla MRI system (12-channel head matrix Rx-coil; Siemens, Erlangen, Germany). For VBM analyses, we obtained T1-weighted high-resolution anatomical images using a three-dimensional magnetization-prepared rapid acquisition with gradient echo [30,31] sequence. The following parameters were used: Repetition time (TR) = 1900 ms; echo time (TE) = 2.26 ms; time of inversion (TI) = 90 ms; bandwidth = 200 Hz/Px. One hundred and seventy-six slices (sagittal orientation) with a slice thickness of 1 mm and a voxel resolution of 1×1×1mm were acquired. The field of view was 256 mm, flip angle was 9 degrees, and the phase encoding direction was anterior–posterior. Acquisition time (TA) was 4:26 min.

For diffusion tensor imaging (DTI) based analysis of white matter, we used an echo-planar imaging (EPI) 2D sequence with the TR 7300 ms and TE 90 ms. TA was 9:44 min. We acquired 56 slices (sagittal orientation) with a thickness of 3 mm per slice. Voxel resolution was 2.5 × 2.5 × 2.5 mm. field-of-view (FOV) was 320 mm and phase encoding was directed anterior to posterior. Diffusion mode was MDDW (multidirectional diffusion weighting). Bandwidth was 1502 Hz/Px, with an EPI factor of 128. For every subject, 2 × 30 diffusion-weighted images along 30 nonparallel directions with *b* = 1000s/mm^2^ and four non-diffusion weighted images with *b* = 0 s/mm^2^ were acquired. The two sets of diffusion-weighted images were merged into one data set for the data analyses.

### Voxel-based morphometry of T1-weighted images

#### Preprocessing

For VBM preprocessing, we used the Computational Anatomy Toolbox (CAT12) toolbox (r1450, Gaser, Structural Brain Mapping group, Jena University Hospital, Jena, Germany; http://www.neuro.uni-jena.de/cat/) for SPM12 (r7487, Statistical Parametric Mapping, Institute of Neurology, London, UK) running under MATLAB (version 2017A, The MathWorks, Inc., Natick, MA).

All images passed a visual quality check for image quality and possible movement artifacts, as well as the homogeneity control. The used MRI images of this study have 1 mm isotropic voxels, so that the extraction of gray matter is not excessively confounded by the voxels containing different tissue types.

Spatial normalization was applied to the individual brain images. For this, we registered all MRI images onto a Montreal Neurological Institute template, making the differences between the image and the template minimal. Finally, we applied spatial smoothing using an isotropic Gaussian kernel of 8 mm full width at half maximum.

#### Statistical analysis

We set up a general linear model (GLM) for the sum score as well as each subscale of the AQ including age and sex as nuisance regressors. Also, the total intracranial volume was used as a covariate to account for different brain sizes. Using linear multiple regression models, we chose a family-wise error (FWE)-corrected threshold of *p* < 0.05 at cluster-level using an initial peak threshold of *p* < 0.001 uncorrected.

In addition, we also performed a quadratic multiple regression analysis for the sum score and each subscale as well, using the function cat_stat_polynomial(x,2) provided by CAT12 with x being the questionnaire data. These values were then included in the GLM as dependent regressors.

For atlas labeling of the clusters, we used the Neuromorphometrics atlas (Neuromorphometrics, Inc.; http://Neuromorphometrics.com) in SPM12 (r7487, Statistical Parametric Mapping, Wellcome Trust Centre for Neuroimaging, London, UK).

### Diffusion tensor imaging

#### Preprocessing

For preprocessing and analyzing the data, we used Tract-Based Spatial Statistics (TBSS) [32] as part of the software package FMBRI Software Library (FSL) [33].

The TBSS preprocessing pipeline started by calculating a brain mask by means of the Brain Extraction Tool [34], using a fractional intensity threshold mask that was laid over the cross-section images of each participant’s MRI data. Eddy current artifacts as well as motion-based artifacts were mathematically corrected [35]. We then performed a visual quality check to determine possible exclusion criteria such as motion artifacts or major brain anomalies. Lastly, the diffusion tensors were calculated, and FA, mean diffusivity (MD), radial diffusivity (RD), and axial diffusivity (AD) values were extracted. We chose these metrics as the most frequently applied parameters of white matter integrity [36]. FA is often used as a global measure of microstructural integrity of white matter tracts, which is sensitive to multiple effects ranging from fiber orientation, packing, and myelination, while RD (which reflects diffusivity perpendicular to the axon) is more sensitive to differences or changes in myelination, and AD (reflecting diffusivity along axonal tracts) is higher for fibers with large axonal diameter and myelination, but normally not affected by demyelination; and finally MD being a more global indicator of white matter integrity [37,38]. These parameters are thus complementary in tracking both pathologies as well as subtle neurodevelopmental processes [39].

Nonlinear registration of all FA, MD, RD, and AD images was applied into a 1×1×1mm standard space. Next, the mean image for each parameter was created and skeletonized. Then, all subjects’ FA/RD/MD and AD data were projected onto the parameters’ mean skeleton.

#### Statistical analysis

Separate GLMs for the sum score as well as each subscale of the AQ were set up and we inserted the 4D projected data into each GLM using a threshold of >0.2. The questionnaire data were used as additional covariate in a single-group average design, while age and sex were included as nuisance regressors. Negative and positive contrasts of the scales were tested with FA, MD, RD, and AD using threshold-free-cluster-enhancement. We used the FSL *randomize* script to run the analysis and the contrasts were tested with 5000 permutations. We chose a tract-based analysis approach, determining the significance level at *p* < 0.05 FWE-corrected threshold.

We prepared illustrations using the Johns Hopkins University (JHU) DTI 81 white-matter labels as well as the JHU white-matter tractography atlas for anatomical labeling in FSL.

## Results

### Voxel-based morphometry

For the linear analysis, we found that the *AQ attention to detail* subscale score was associated with higher GMV in the right pre- and postcentral gyrus (*k* = 1093 voxels, x/y/z (mm) = 28/−21/62, T = 3.124, *p* = 0.019 FWE cluster-level corrected).

We found nonlinear associations (using the quadratic multiple regression design) of a higher *AQ imagination* subscale score and increased GMV in the bilateral calcarine cortex and the bilateral lingual gyrus (*k* = 1026 voxels, x/y/z (mm) = −12/−86/−2, *T* = 3.124, *p* = 0.024 FWE cluster-level corrected). We also found that a higher *AQ imagination* subscale score was associated with higher GMV in the left superior frontal gyrus and the left middle frontal gyrus (*k* = 965 voxels, x/y/z (mm) = −30/42/24, *T* = 3.124, *p* = 0.030 FWE cluster-level corrected).

### Diffusion tensor imaging

#### Fractional anisotropy

For the *AQ attention switching* subscale, we found a negative correlation with FA values in the bilateral uncinate fasciculus as well as the bilateral inferior fronto-occipital fasciculus (details can be found in Table 1). We also found a correlation with lower FA values in the left hippocampal cingulum (see Table 1).

**Table 1.** Associations of AQ subscales with DTI FA values (TBSS, thresholded at *p* < 0.05, FWE cluster-level corrected).

FA positive correlations				
Subscale	White matter tract	*k* (voxels)	*p* ^a^	MAX x/y/z (mm)^b^
*AQ communication*	Left cingulum (cingulate gyrus)	53	0.039	−12/25/18
		19	0.046	−13/16/25

FA negative correlations				
Subscale	White matter tract	*k* (voxels)	*p* ^a^	MAX x/y/z (mm)^b^
*AQ attention switching*	Left uncinate fasciculus	649	0.003	−30/11/−5
	Right uncinate fasciculus	790	0.001	22/24/−9
	Left inferior fronto-occipital fasciculus	590	0.008	−30/12/−6
	Right inferior fronto-occipital fasciculus	925	0.003	23/24/−5
	Left hippocampal cingulum	49	0.033	−22/−29/−16

Abbreviations: AQ, autism spectrum quotient; DTI, diffusion tensor imaging; FA, fractional anisotropy; MNI, Montreal Neurological Institute; TBSS, tract-based spatial statistics.

a FWE cluster-level corrected.

b MNI coordinates.

**Figure 1.**
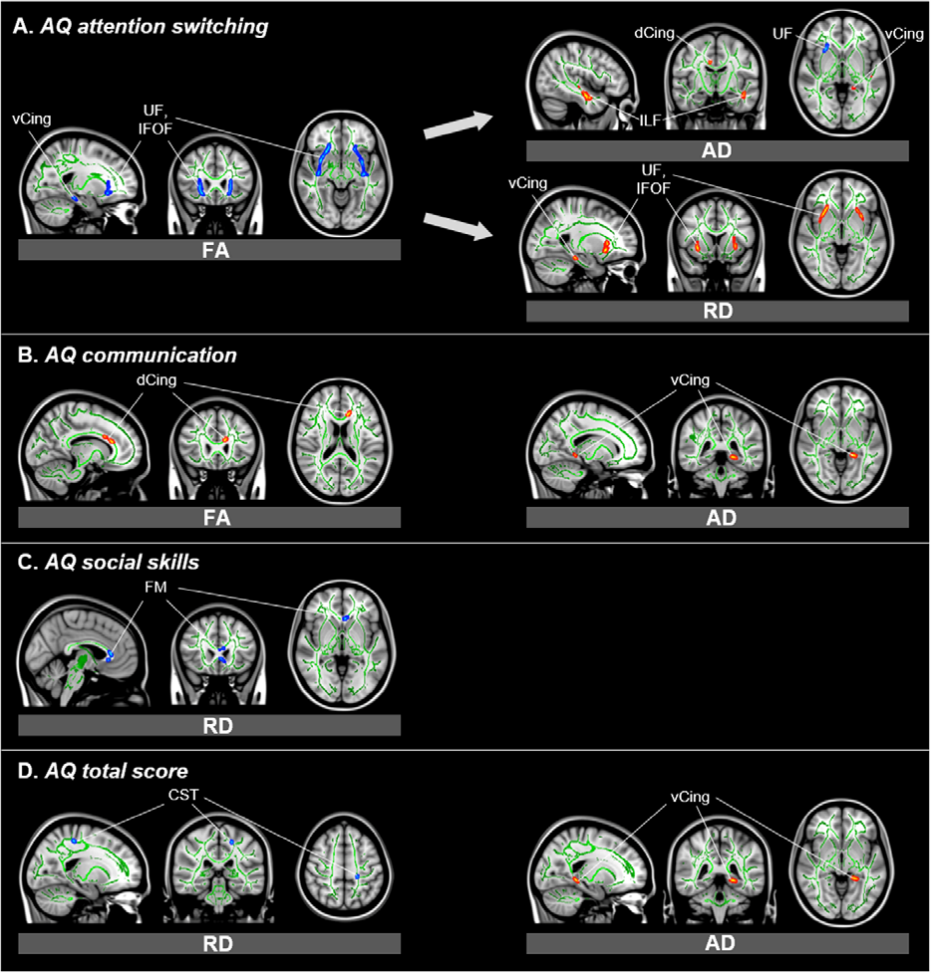
Associations of AQ subscales with DTI parameters (TBSS, thresholded at *p* < 0.05, FWE cluster-level corrected). Images are displayed in radiological (left corresponding to right side) orientation. Abbreviations: AD, Axial Diffusivity; AQ, Autism Spectrum Quotient; CST, Cerebrospinal Tract; dCing, Dorsal Cingulum; DTI, Diffusion Tensor Imaging; FA, Fractional Anisotropy; FM, Forceps Minor; FWE, Family-Wise Error; IFOF, Inferior Fronto-Occipital Fasciculus; ILF, Inferior Longitudinal Fasciculus; RD, Radial Diffusivity; TBSS, Tract-Based Spatial Statistics; UF, Uncinate Fasciculus; vCing, Ventral Cingulum.

**Figure 2.**
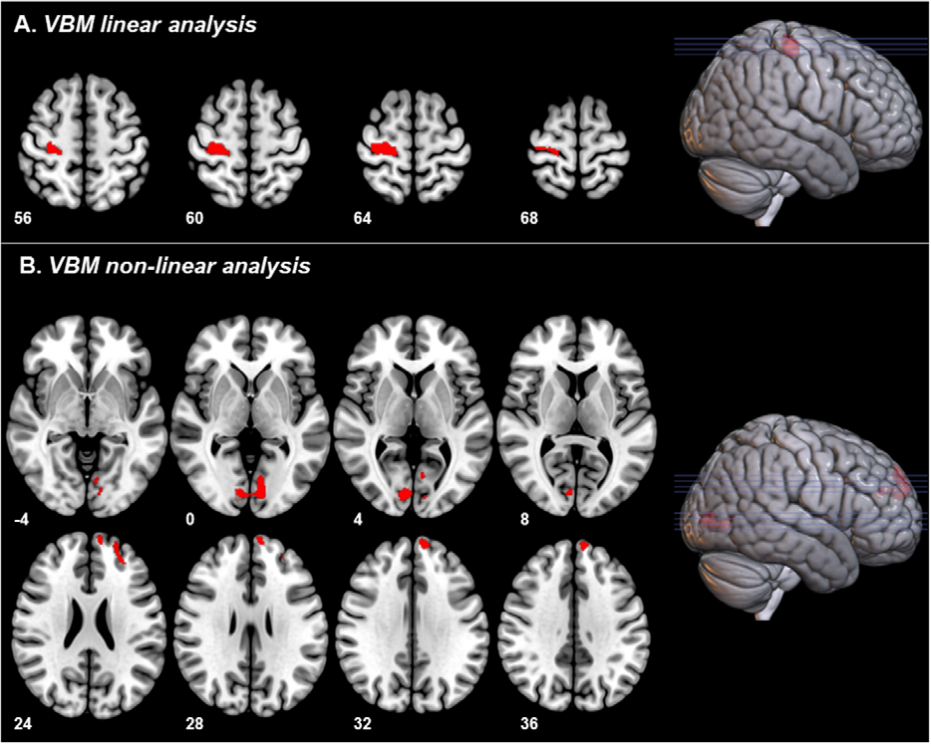
Results of voxel-based morphometry analyses (*p* < 0.05, FWE cluster-level corrected, initial threshold *p* < 0.001 uncorr.). Images are displayed in radiological (left corresponding to right side) orientation. (A) Linear analysis: Association between *autism spectrum quotient (AQ) attention to detail* subscale and gray matter volume (GMV). (B) Nonlinear analysis: Association between *AQ imagination* subscale and GMV. Abbreviation: VBM, voxel-based morphometry.

A positive correlation between FA values and the *AQ communication* subscale was found in the left dorsal cingulum (see Table 1).

#### Axial diffusivity

For the *AQ total* score, we found a positive correlation with AD in the left hippocampal cingulum (see Table 2). The *AQ attention switching* subscale score was associated with higher AD in the right cingulum (cingulate gyrus), in the left hippocampal cingulum, as well as in the left inferior longitudinal fasciculus (see Table 2).

**Table 2.** Associations of AQ subscales with DTI AD values (TBSS, thresholded at *p* < 0.05, FWE cluster-level corrected).

AD positive correlations				
Subscale	White matter tract	*k* (voxels)	*p* ^a^	MAX x/y/z (mm)^b^
*AQ total score*	Left hippocampal cingulum	83	0.004	−19/−41/−5
*AQ attention switching*	Right cingulum (cingulate gyrus)	42	0.017	9/−19/35
	Left hippocampal cingulum	43	0.016	−19/−41/−5
	Left inferior longitudinal fasciculus	142	0.020	−44/−12/−18
*AQ communication*	Left hippocampal cingulum	83	0.002	−16/−40/−5

AD negative correlations				
Subscale	White matter tract	*k* (voxels)	*p* ^a^	MAX x/y/z(mm)^b^
*AQ attention switching*	Right uncinate fasciculus	52	0.033	28/20/0

Abbreviations: AD, axial diffusivity; AQ, autism spectrum quotient; DTI, diffusion tensor imaging; MNI, Montreal Neurological Institute; TBSS, tract-based spatial statistics.

a FWE cluster-level corrected.

b MNI coordinates.

A negative correlation for the *AQ attention switching* subscale score and AD was found in the right uncinate fasciculus (see Table 2).

The AQ communication subscale score was associated with higher AD values in the left hippocampal cingulum (see Table 2).

#### Mean diffusivity

The *AQ attention switching* subscale score correlated positively with MD values in the right cingulum (cingulate gyrus), in the left inferior longitudinal fasciculus, and in the left uncinate fasciculus (see Table 3).

**Table 3.** Associations of AQ subscales with DTI MD values (TBSS, thresholded at *p* < 0.05, FWE cluster-level corrected).

	MD positive correlations			
Subscale	White matter tract	*k* (voxels)	*p* ^a^	MAX x/y/z (mm)^b^
*AQ attention switching*	Right cingulum (cingulate gyrus)	29	0.021	9/−19/35
	Left inferior longitudinal fasciculus	154	0.027	−38/−8/−20
		23	0.040	−45/−8/−19
	Left uncinate fasciculus	83	0.029	−37/−8/−20

Abbreviations: AQ, autism spectrum quotient; DTI, diffusion tensor imaging; MD, mean diffusivity; MNI, Montreal Neurological Institute; TBSS, tract-based spatial statistics.

a FWE cluster-level corrected.

b MNI coordinates.

#### Radial diffusivity

The *AQ total* score was associated with lower RD values in the left corticospinal tract.

The *AQ attention switching* subscale score showed a positive correlation with RD values in the left hippocampal cingulum, in the right inferior fronto-occipital fasciculus, and in the bilateral uncinate fasciculus.

The *AQ social skills* subscale score correlated positively with RD values in the forceps minor (details can be found in Table 4).

**Table 4.** Associations of AQ subscales with DTI RD values (TBSS, thresholded at *p* < 0.05, FWE cluster-level corrected).

	RD positive correlations			
Subscale	White matter tract	*k* (voxels)	*p* ^a^	MAX x/y/z (mm)^b^
*AQ attention switching*	Left hippocampal cingulum	21	0.022	−25/−28/−19
	Right inferior fronto-occipital fasciculus	271	0.027	29/16/−4
	Left uncinate fasciculus	242	0.027	−30/11/−4
		223	0.019	−37/−8/−20
	Right uncinate fasciculus	479	0.009	30/14/−4
*AQ social skills*	Forceps minor	38	0.043	−3/23/−2
		27	0.042	−7/25/14
		25	0.045	−3/27/8

RD negative correlations				
Subscale	White matter tract	*k* (voxels)	*p* ^a^	MAX x/y/z (mm)^b^
*AQ total score*	Left corticospinal tract	12	0.047	−19/−32/51

Abbreviations: AQ, autism spectrum quotient; DTI, diffusion tensor imaging; MNI, Montreal Neurological Institute; RD, radial diffusivity; TBSS, tract-based spatial statistics.

a FWE cluster-level corrected.

b MNI coordinates.

## Discussion

Our findings combine gray and white matter characterization of neural correlates of subclinical autistic-like traits. They support a subclinical continuum toward effects seen in clinical ASD, as seen in gray matter effects in prefrontal and primary visual cortices and white matter effects seen in the uncinate fasciculus [40,41]. Within our exploration of facet-level phenotypes, the AQ attention switching subscale showed most associations with brain structure, including higher AQ *attention switching* subscale scores correlating with lower FA values in the bilateral uncinate fasciculus as well as the bilateral inferior fronto-occipital fasciculus. The AQ *attention switching* subscale score was also associated with higher RD values in the IFOF and the bilateral uncinate fasciculus.

Higher-level visual processing such as face recognition and recognition of the facial expression of emotion is strongly linked to the bilateral IFOF and the bilateral uncinate fasciculus that constitute a network between the occipital and the orbitofrontal cortex [42,43]. Since patients with ASD show impairment in recognizing facial expressions [44], the IFOF may play an important role in the neurobiology of ASD [45].

Our results partly overlap with previous models from studies focusing on ASD patients that showed significantly decreased FA values in the bilateral IFOF, the bilateral uncinate fasciculus, the cingulate cingulum, and the right hippocampal cingulum as well as in the bilateral inferior longitudinal fasciculus, superior longitudinal fasciculus, and the right forceps major and minor [41,46]. In a prior study encompassing healthy adults, the AQ score was also correlated with FA alterations located on the IFOF [20]. This may show a neurobiological continuum of autistic-like traits at least for the IFOF and uncinate fasciculus. It has also been repeatedly shown that there seems to be a greater impairment in the left hemisphere in patients with ASD [47]. This is in line with our findings of altered white matter integrity in nonclinical individuals.

There are only few association studies for the nonclinical population, mostly limited in sample size or in use of a single modality. Interestingly, our findings differ from some of these previous studies in which a higher AQ *total score* correlated positively with FA values in the left inferior longitudinal fasciculus [19]. Another study regarding healthy adults showed a correlation between the volume of the white matter pathway between the STS and AMG with the AQ total score [21]. Other studies showed no associations between AQ scores and brain structure, including GMV and FA values, in the general population [48]. However, Koolschijn et al. used the short version of the AQ (28 items), whereas we used the long version (50 items), making direct comparison between the studies limited. Furthermore, recent psychometric analysis of the AQ does not support the use of total-scale scores as there is strong evidence for divergence between the factors of the AQ [49].

On the behavioral level, the AQ’s social attention subscales, including AQ attention switching, have been associated with poorer face recognition in a nonclinical sample [50], biological motion perception [51], and also poorer visual working memory performance [52], while higher attention to detail scores are linked to better visual working memory performance [52]. Also, AQ attention switching is associated with facial stimulus gaze-cueing [53]. Our findings might therefore represent structural correlates of socially relevant dysfunctions within the nonclinical spectrum.

A second main finding of our study is a more detailed analysis of the nature of white matter changes associated with subclinical traits. Prior studies showed that changes in AD and RD values may be used to differentiate myelin loss versus axonal injury [54]. We were able to show that the decreased white matter integrity in subjects with higher AQ scores may be caused by a mixture of variation of myelinization and axonal variations. RD values show the diffusion perpendicular to the axon and are therefore majorly affected by myelinization while AD values represent the diffusion along the axon and may show axon variations [55]. Our study shows that higher *AQ attention switching* subscale scores are associated with increased MD and RD values in the uncinate fasciculus, while there is a negative correlation with AD values in the uncinate fasciculus. Therefore, the altered white matter integrity within the uncinate fasciculus seems to be caused by a combination of both variation of myelinization and axonal variation. For the left hippocampal cingulum, however, we found a negative correlation between the *AQ attention switching* subscale score and FA values, but a positive association for both AD and RD values. This may show that the decreased white matter integrity in the hippocampal cingulum is caused only by variation of myelinization and not axonal damage.

Our findings support similar subclinical continuum models for GMV. Major anatomical findings were that a higher AQ *attention to detail* subscale score was associated with increased GMV in the right pre- and postcentral gyrus, and the AQ *imagination* subscale score correlated positively with GMV in the bilateral calcarine cortex, the left lingual gyrus, the left superior frontal gyrus, as well as the left middle frontal gyrus. These results resemble regions found in previous case–control studies, showing increased GMV in the right precentral gyrus and left superior frontal gyrus in subjects with ASD [40]. The left superior frontal gyrus and the precentral gyrus play an important role in movement sequences [56] and working memory [57]. Both, movement sequencing and working memory, are reportedly impaired in patients with ASD [58,59].

Our results show that there are neurobiological associations with autistic-like traits in a nonclinical sample, including altered white matter integrity as well as GMV. Also, we found striking resemblance to previous findings in patients with ASD. Therefore, our study shows that correlations with biological parameters such as in ASD patients are also present to a lesser extent in the nonclinical subjects.

This study must be interpreted in the context of some limitations. First, our sample consisted of mostly females, while a strong male bias has been reported in ASD prevalence [60]. This may have caused our sample to score lower in the AQ than the general population. Our sample showed a mean of 13.14 for the *AQ total* score with an SD of 5.11 while previous systematic reviews of the AQ in a nonclinical population reported a mean of 16.94 (95% CI 16.4–17.4), but for females, a mean of 14.88 (95% CI 13.3–16.5) was found [61], while internal consistency of some AQ subscales was low, consistent with previous research.

Also, there are age-related differences in white matter integrity as well as GMV [62,63]. Our sample showed a mean age of only 23.9 (SD = 3.9). Even while controlling for age, our study has the advantage of limiting the variance of the brain structure caused by age differences because of the young sample with a narrow age range. Since our study is limited to nonclinical subjects, future studies might combine the approach of a broader range of clinical and subclinical subjects.

Third, as we did not include ASD subjects, we did not cover the full spectrum but nonclinical phenotype expressions cannot infer to brain structural changes seen in ASD. This is relevant not only given some inconsistencies of AQ factor structure across populations [49]. Neither the latent structure of autistic-like traits (tapped by the AQ) [64] nor that of neurobiological features [65] are clarified.

## Conclusion

This study demonstrates an association of brain structural variation with an extended autistic trait phenotype, including key areas and fiber tracts relevant to ASD pathology. This emphasizes the importance of considering autistic traits and their neurobiological underpinnings on a dimensional spectrum, and of differentiating between particular phenotype facets with nonoverlapping (or even diametrically different) neural substrates.

## Data Availability

The data that support the findings of this study are available from the authors/senior author. Restrictions apply to the availability of these data.
